# Growth hormone and aging: a clinical review

**DOI:** 10.3389/fragi.2025.1549453

**Published:** 2025-04-07

**Authors:** Luis E. Fernández-Garza, Fernando Guillen-Silva, Marcos A. Sotelo-Ibarra, Andrés Ely Domínguez-Mendoza, Silvia A. Barrera-Barrera, Hugo A. Barrera-Saldaña

**Affiliations:** ^1^ Innbiogem SC, Monterrey, Mexico; ^2^ Servicio de Medicina Interna, Hospital General de Zona No. 2, Instituto Mexicano del Seguro Social, Monterrey, Mexico; ^3^ LANSEIDI-CONAHCyT, Monterrey, Mexico; ^4^ Facultad de Ciencias Biológicas de la Universidad Autónoma de Nuevo León, Ciudad Universitaria, San Nicolás de Los Garza, Mexico; ^5^ Facultad de Medicina de la Universidad Autónoma de Nuevo León, Monterrey, Mexico; ^6^ Dirección de Investigación Científica de Laboratorios Columbia, Coyoacán, Mexico

**Keywords:** human growth hormone, insulin-like growth factor I, human growth hormone deficiency, anti-aging treatments, aging

## Abstract

Aging is a complex biological process characterized by functional decline, reduced quality of life, and increased vulnerability to diseases such as type 2 diabetes, cardiovascular conditions, neurodegeneration, and cancer. Advances in medical technology have introduced the concept of aging therapies, with growth hormone (GH) and its primary mediator, insulin-like growth factor 1 (IGF-1), receiving considerable attention for their potential to counteract age-related physiological and metabolic changes. GH plays a multifaceted role in the human body, primarily influencing body composition by increasing muscle mass, reducing fat tissue, promoting bone formation, and regulating the metabolism of proteins, lipids, and glucose. Additional effects have been noted on endothelial function, cognitive performance, and circadian rhythms. This review examines the molecular mechanisms of GH in aging, its potential as an anti-aging therapy, and findings from clinical trials involving these hormones for this purpose. It also addresses the associated adverse effects, limitations, and controversies. While some studies report significant benefits, these therapies’ long-term safety and efficacy in promoting healthy aging remain uncertain, highlighting the need for further research.

## 1 Introduction

Human aging is an incredibly complex process marked by time-dependent functional decline that lowers the quality of life (Cai et al., 2022). Furthermore, aging is the greatest risk factor for several diseases, including type 2 diabetes (T2D), cardiovascular diseases (CVD), neurodegeneration, and cancer (Duran-Ortiz et al., 2021). Nevertheless, a trend has been seen that as medical technology has advanced, the average lifespan has greatly increased globally.

In medical technology, aging therapies focus on preventing and treating illnesses that become more prevalent with age. These therapies may aim to slow or halt age-related changes after they have occurred or work proactively to delay or prevent them at different stages of life. A major concern is that a therapy effective for one of these objectives may be ineffective—or even counterproductive—for another(s) of these age-related changes (Hadley et al., 2005).

The endocrine system is the most relevant in aging. There is considerable evidence that the actions of GH and IGF-1 play key roles throughout the lifespan and during aging in mammals (Bartke, 2022). Our review aims to define the physiological mechanisms of human GH (HGH) involvement in aging, the existing scientific evidence on how it translates into human anti-aging therapies, and the adverse effects, limitations, and controversial aspects surrounding them.

## 2 Growth hormone as a key regulator of growth and metabolism

HGH, also known as somatotropin, is located on chromosome 17 and its gene is structured into five highly conserved clusters. It is expressed mainly in two types of tissues: the *hGH-N* gene only in somatotropic cells of the pituitary gland, while the human chorionic somatomammotropin hormone (CSH) A (*hCS-A*) and human CSH B (*hCS-B*) genes are expressed in placental trophoblasts (Chen, et al., 1989). HGH is a single polypeptide chain-globular protein composed of 191 amino acids, 22 kDa (kDa) of molecular weight, containing two disulfide bridges, and lacks sugar residues (Brinkman et al., 2024; Pérez-Ibave et al., 2019).

**FIGURE 1:**
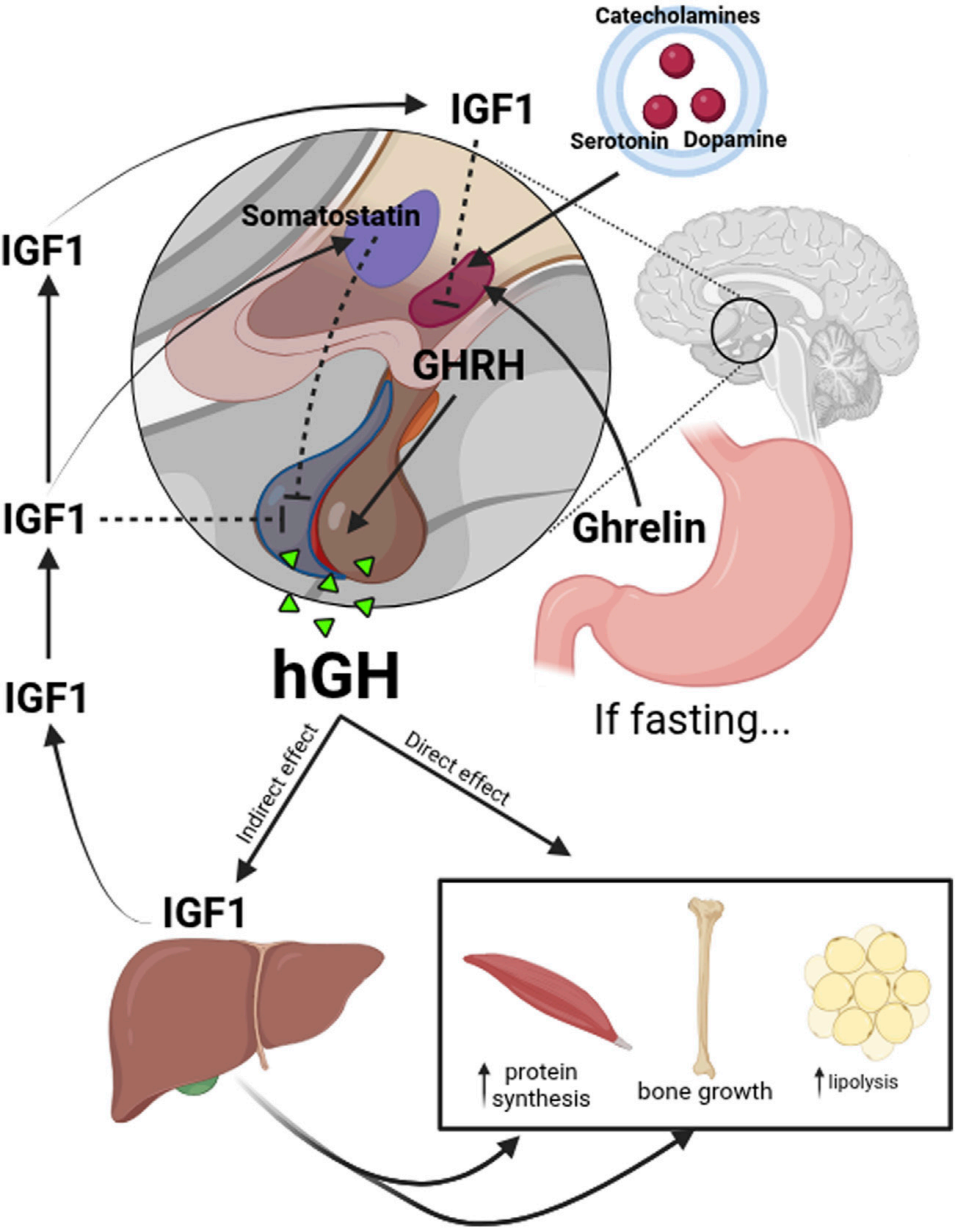
Upstream and downstream regulation of HGH. The release of HGH is regulated by multiple signals in the hypothalamus. GHRH secreted by neurons in the arcuate nucleus, stimulates HGH production in the anterior pituitary gland. In contrast, somatostatin, secreted by the periventricular nucleus of the hypothalamus, inhibits HGH release. Factors such as catecholamines, serotonin, and dopamine modulate GHRH secretion, while ghrelin, primarily secreted by the stomach during fasting conditions, enhances HGH release. HGH directly affects various tissues, stimulating protein synthesis in muscles, lipolysis in adipose tissue, and chondrocyte differentiation in bones. It also acts indirectly through IGF-1, which is primarily produced in the liver and mediates bone growth and anabolic metabolism. IGF-1 also has a direct effect in the modulation of GHRH secretion inhibiting the arcuate nucleus cells and the production of HGH in the pituitary gland; IGF-1 stimulates the production of somatostatin.

Multiple signals in the hypothalamus regulate the release of HGH. Growth Hormone-Releasing Hormone (GHRH), secreted by neurons in the arcuate nucleus of this brain structure, stimulates HGH production in the anterior pituitary gland. In contrast, somatostatin, secreted by the periventricular nucleus, inhibits HGH release. Factors such as catecholamines, serotonin, and dopamine modulate GHRH secretion, while ghrelin, primarily secreted by the stomach during fasting conditions, enhances HGH release. HGH exerts direct effects in various tissues, stimulating protein synthesis in muscle, lipolysis in adipose tissue, and chondrocyte differentiation in bones. It also acts indirectly through IGF-1, which is primarily produced in the liver and mediates bone growth and anabolic metabolism. IGF-1 also has a direct effect in modulating the secretion of GHRH by inhibiting the arcuate nucleus cells; the production of HGH and IGF-1 stimulates the production of somatostatin (Figure 1).

The main function of HGH is stimulating linear growth in children by acting directly and indirectly via IGF-1 on the epiphyseal plates of long bones, resulting in increased chondrocyte proliferation. Regarding adults, HGH has other described functions, including increasing lipolysis and lipid oxidation, which leads to mobilization of stored triglycerides, stimulation of protein synthesis that includes skeletal muscle anabolism, antagonism of insulin action that leads to glucose intolerance, and decrease of phosphate, water, and sodium renal excretion (Chikani and Ho, 2013; Ho et al., 2023).

HGH exerts its effects through the JAK-STAT signaling pathway, influencing growth and metabolism across various tissues and organs, particularly cartilage and bone development. Metabolically, HGH modulates both insulin and IGF-1 production. Abnormalities in HGH secretion or function are implicated in multiple diseases. In adults, excessive HGH secretion—most commonly from a pituitary adenoma—leads to acromegaly, whereas excess HGH secretion occurring before the closure of epiphyseal growth plates results in gigantism. On the other hand, insufficient HGH levels during childhood cause dwarfism, and HGH deficiency in adults is associated with reduced skeletal muscle mass, increased visceral adiposity, and a range of secondary health issues such as cardiovascular disease, mood disturbances (including depression and anxiety), and a notable lack of energy (Brinkman et al., 2024).

**FIGURE 2:**
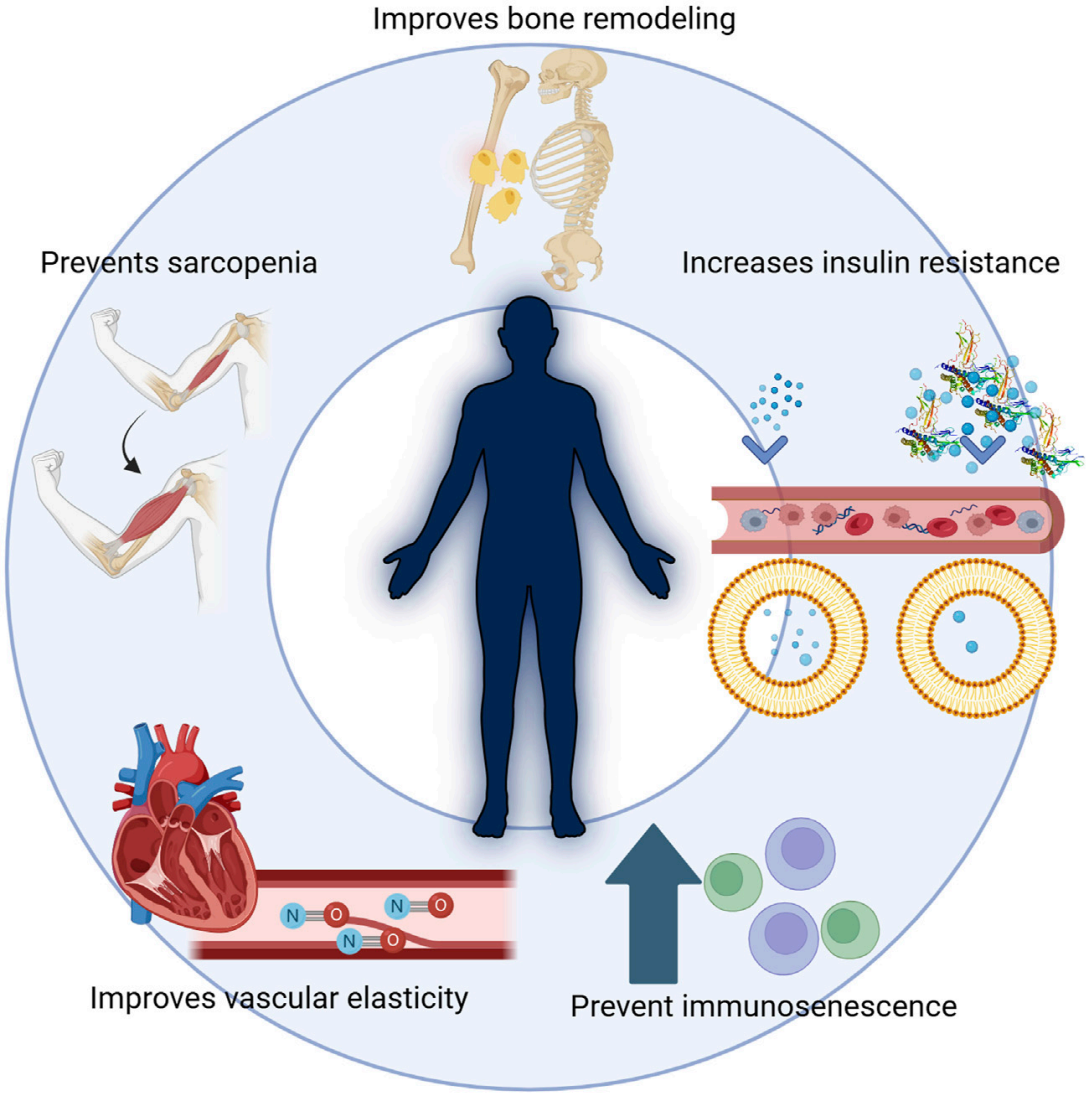
HGH and its multiple functions in the body. Beyond promoting growth during childhood and adolescence, HGH maintains homeostasis, supports body composition, and sustains overall metabolic balance and well-being. HGH is critical in improving bone remodeling, preventing sarcopenia, and enhancing vascular elasticity. Additionally, it influences insulin resistance, impacting metabolic balance, and helps prevent immunosenescence, thus supporting overall health during aging. While it continues to influence numerous organs and systems throughout adulthood, its effects gradually diminish as individuals advance into older age.

Beyond promoting growth during childhood and adolescence, HGH maintains homeostasis, supports body composition, and sustains overall metabolic balance and wellbeing (Figure 2). While it continues to influence numerous organs and systems throughout adulthood, its effects gradually diminish as individuals advance into older age.

### 2.1 Recombinant human growth hormone

The therapeutic use of HGH dates back to the late 1950s. Originally, its use was limited to treating children with a complete deficiency of this hormone, and until the mid-1980s, it was obtained from the pituitary glands of cadavers (Ayyar, 2011).

Although these preparations were biologically active, their production was limited due to the scarcity of this natural source. In 1985, the first reports emerged of Creutzfeldt-Jakob disease in individuals who had received this version of HGH during childhood and adolescence. This disease is characterized by ataxia and a progressive neurological syndrome and is caused by an unconventional agent known as a prion protein. More than 50 cases of Creutzfeldt-Jakob disease have been reported in individuals treated with hormones derived from cadavers. For this reason, the use of HGH obtained from this source in humans was banned in late 1985 (Barrera-Saldaña et al., 2019; Uttley et al., 2020).

In the mid-1980s, advances in biotechnology made it possible to produce unlimited quantities of HGH through genetic engineering or recombinant DNA methods. This biosynthetic or recombinant HGH (hereafter simply commonly known as HGH) is biologically active and free from contaminants. The ability to produce HGH in large quantities and the improved understanding of its metabolic effects have expanded and diversified its potential therapeutic applications (Barrera-Saldaña, 1998; Barrera-Saldaña et al., 2007).

### 2.2 Aging and the endocrine system

Aging is characterized by changes in all biological systems, and the endocrine system is not an exception. During aging, the secretory pattern of hormones produced by the hypothalamic-pituitary axis changes, as does its sensitivity to negative feedback by end hormones, such as the thyroid hormones (T3 and T4), cortisol, estradiol, testosterone, and IGF-1 (van den Beld et al., 2018). This axis includes the secretion of HGH into the circulation, and the consequent stimulation of IGF-1 secretion (van den Beld et al., 2018). Somatopause is a gradual and progressive decline in HGH secretion that occurs normally with aging and is associated with increased adipose tissue (Di Somma et al., 2011). The age-related changes are considered to be similar to those observed in HGH deficiency, and from this point is where the controversy begins, as it is observed, at least in model animals, that this deficiency or resistance has points in favor of prolonging life expectancy.

Several mutations that impair the somatotropic axis have been associated with increased lifespan in mice. Notably, mutations in PROP1 (Prop1df/df, Ames dwarf mice) and POU1F1 (Pou1f1dw/dw, Snell dwarf mice) disrupt anterior pituitary development, leading to deficiencies in GH, thyroid-stimulating hormone (TSH), and prolactin (PRL), significantly extending lifespan (Junnila et al., 2013). Similarly, mice with a GHR deletion (Ghr−/−), which models Laron syndrome (characterized by low levels of IGF-1), exhibit increased longevity and metabolic benefits. Additionally, mice heterozygous for IGF-1 receptor (IGF-1R) deletion (*Igf1r*+/−) show extended lifespan, particularly in females, while lit/lit mice, mutated in GHRH, also display longevity advantages. However, although similar genetic variations have been identified in humans, their impact on longevity remains uncertain (Junnila et al., 2013). Another example is the observation that older patients with Laron syndrome exhibit brain structure and function similar to that of younger, unaffected individuals, raising the question of whether inhibition of HGH/IGF1 axis might have a potential protective effect against age-related cognitive decline. Notably, individuals with Laron syndrome also exhibit an almost complete absence of cancer, further suggesting that reduced IGF-1 signaling may confer protective effects against both neurodegeneration and tumorigenesis (Nashiro et al., 2017). Interestingly, this inverse relationship between IGF-1 and longevity is not unique to humans; similar findings have been reported in other species. In dogs, for example, low IGF-1 levels are strongly associated with smaller body size and increased lifespan, whereas larger breeds with higher IGF-1 levels tend to exhibit shorter lifespans and accelerated aging (Sutter et al., 2007; Kraus et al., 2013). These observations across species support the idea that modulating GH/IGF-1 signaling plays a critical role in aging and longevity.

### 2.3 Muscular mass

IGF-1 is a primary mediator of muscle repair and growth, stimulated by GH in the liver and via its paracrine-autocrine secretion in muscle (Sattler, 2013). The human skeletal muscle IGF-1 gene can be cleaved to produce three IGF-1 subtypes, IGF-1Ea, IGF-1Eb, and IGF-1Ec, this last one is also called mechanical growth factor (MGF). Muscle damage by external factors can induce the expression of the IGF-1 isoform MGF, followed by the appearance of calcium-dependent cell adhesion molecules and satellite cell marker, mucin. The peak of the MGF expression level is considered to trigger muscle satellite cells (SCs) activation. Therefore, the level of SC reserve determines the potential for muscle regeneration (Bian et al., 2020). With aging, the compliance of the skeletal muscle may decline and is accompanied by a reduction in the ability to synthesize and secrete MGF.

Sarcopenia is a progressive, age-related disease characterized by the loss of muscle mass and function, affecting all older individuals at some point in life. Beyond its direct impact on mobility and quality of life, sarcopenia is associated with frailty, social isolation, depression, and an increased risk of disability and mortality (Sattler, 2013). Multiple factors contribute to its development, including lifestyle, exercise habits, comorbidities, and hormonal dysregulation (Bian et al., 2020). Among the endocrine regulators, the decline in GH and IGF-1 signaling plays a crucial role, particularly the MGF isoform of the latter, which is involved in muscle regeneration and repair (Dai et al., 2010; Bian et al., 2020). Studies indicate that reductions in IGF-1 and MGF levels correlate significantly with decreased skeletal muscle mass and function in older populations (Bian et al., 2020).

The European Working Group on Sarcopenia in Older People (EWGSOP2) recently updated the diagnostic criteria for sarcopenia, emphasizing low muscle strength as a primary indicator, along with decreased muscle quantity or quality, and impaired physical performance (Cruz-Jentoft et al., 2019). Given its multifactorial etiology, sarcopenia has been the subject of two major international consensus statements (2010 and 2019), which have refined its algorithm diagnosis and its categories (Cruz-Jentoft et al., 2019). Although these consensuses do not describe much about its management, the International Clinical Practice Guidelines for Sarcopenia (ICFSR) published in 2018, as part of pharmacological interventions, names HGH as a drug that could increase muscle mass associated with nitrogen retention, however, without improvement in muscle strength (Dent et al., 2018).

### 2.4 Adipose tissue and lipid metabolism

The effects of HGH and IGF-1 on lipid metabolism are due to a complex interaction with other hormones controlling substrate metabolism. HGH is lipolytic and releases free fatty acids (FFA) mainly from visceral adipose tissue and less so from subcutaneous fat by increasing the activation of hormone-sensitive lipase (HSL) in adipocytes by different mechanisms, the most recognized is via enhancement of agonist-induced stimulation of the β-adrenergic receptors (β-AR) (Vijayakumar et al., 2011). Additionally, HGH has little effect on the uptake of FFAs by the liver via lipoprotein lipase (LPL) and hepatic lipase (Sattler, 2013).

GH also plays a role in adipogenesis, induction of adipocyte differentiation via the activation of STAT5, and its subsequent association with peroxisome proliferator-associated receptor-γ (PPAR-γ). Another important aspect of GH action is its direct repression of glucose uptake or antagonism of insulin signaling in adipose tissue. Therefore, GH maintains triglyceride storage in the liver through three mechanisms, inhibiting intrahepatic triglyceride lipolysis, inhibiting lipid oxidation, and enhancing lipogenesis (Vijayakumar et al., 2011). Lastly, GH stimulates triglyceride uptake into skeletal muscle via LPL for energy during muscle contraction or storage as intramyocellular lipids (Sattler, 2013).

More recently, the surface proteins of lipid droplets (LD) have been studied for their multiple functions, including the regulation of lipolysis. Among these proteins, ATGL, G0S2, PLIN1, and FSP27 stand out, as these are transcriptional targets of the adipogenesis regulator PPAR-γ. It has been seen that direct alterations of these proteins lead to dysregulation in the lipolysis process. It has been described that GH downregulates the expression of FSP27 and G0S2, resulting in the fragmentation of LD, and causing the flow of FFAs to the systemic circulation. However, at the same time, it has been seen that the administration of GH also results in an upregulation of the FSP27 mRNA advising that there is an additional transcription mechanism involved, which suggests that GH maintains adipose tissue homeostasis and not only its degradation (Kopchick, et al., 2019; Sharma et al., 2019).

### 2.5 Glucose metabolism

HGH impacts glucose metabolism due to its effect on insulin sensitivity (IS). GH induces insulin resistance by many different mechanisms known so far. These include increased FFA flux from the adipose tissue to the systemic circulation, possibly leading to insulin resistance (Møller and & Jørgensen, 2009; Sattler, 2013). In addition, GH induced the overexpression of protein of the suppressor of cytokine signaling (SOCS) family, in particular SOCS-1 and SOCS-3, which are well described in their role in insulin resistance and downregulation of insulin signaling (Vijayakumar et al., 2011). Also, GH increases hepatic glucose output by promoting glycogenolysis and gluconeogenesis in the liver (Sattler, 2013). Lastly, GH increases the expression of the p85α regulatory sub-unit of the PI3K, which depends on its amount in the cell that serves as a molecular switch that either enhances or suppresses insulin signaling (Vijayakumar et al., 2011).

These unwanted effects of HGH need to be considered when administering its recombinant version. It is suggested that blood glucose and insulin levels should be monitored during its administration due to the risk of progression to glucose intolerance or the development of T2D, especially in genetically predisposed individuals (Błaszczyk et al., 2023). Also, the effect of HGH on glucose homeostasis has been implicated in the development of other metabolic diseases, such as metabolic dysfunction-associated steatotic liver disease (MASLD). HGH modulates several metabolic pathways that affect fat accumulation in the liver, thereby influencing IS and glucose balance in the body. These pathways include both the direct regulation of lipolysis and the modulation of inflammation and oxidative stress in liver tissue, particularly in the context of aging, when hepatic steatosis and insulin resistance become more prevalent and adversely affect metabolic health (Dichtel et al., 2022).

### 2.6 Role of growth hormone in the aging brain

The role of HGH extends beyond body growth and metabolism, affecting several neuroendocrine and cognitive functions. It plays a key role in central nervous system (CNS) development by regulating neurogenesis, cell differentiation, axonal growth, synaptogenesis, and myelination (Janchevska et al., 2018). In addition, HGH influences synaptic plasticity, which has important implications for memory and learning (Martinez-Moreno and Aramburo, 2020). HGH deficiency has been associated with memory problems, fatigue, sleep disturbances, and attention deficits, which negatively affect individuals’ emotional wellbeing and quality of life (Brod et al., 2014). Regarding aging, declining HGH levels and the density of GH binding sites in the brain may contribute to cognitive decline in the older people (Nyberg and Mathias Hallberg, 2013; Wasinski et al., 2019).

GH leaves the systemic circulation and crosses the blood-brain barrier (BBB) reaching its responsive sites in the brain, including the choroid plexus, hypothalamus, and hippocampus. This has been described in human studies when concentrations of GH and IGF-1 increase in cerebrospinal fluid (CSF) posterior to the GH replacement therapy (Nyberg and Mathias Hallberg, 2013). Nevertheless, it is still controversial whether the action on the brain is triggered by the GH or its mediators IGF-1 or IGF-2.

In animal models, homozygous deletion of IGF-1 or IGF-1R genes results in high lethality and severe CNS deficits, including reduced brain size and loss of specific neurons, highlighting the essential role of IGF-1 signaling in neurodevelopment (Ashpole et al., 2015). However, heterozygous deletion of IGF-1R (Igf1r+/−) does not impair viability while has been associated with increased lifespan and enhanced resistance to oxidative stress, suggesting that partial downregulation of IGF-1 signaling may have beneficial effects on longevity (Holzenberger et al., 2003).

### 2.7 Endothelial function and vascular health

HGH is directly involved in maintaining vascular health, primarily through stimulating nitric oxide (NO) production, a short-lived gas considered an essential vasodilator. IGF-1 allowed endothelial progenitor cells (EPC) to be mobilized from the bone marrow into the bloodstream (Devin et al., 2008). Once in circulation, they are directed to sites of vascular damage contributing to the repair and subsequent regeneration of the endothelium. This signaling cascade is fundamental to avoiding endothelial dysfunction and maintaining blood vessel integrity. The decrease in HGH production during aging is particularly relevant, as it is associated with reduced circulating EPCs in the blood and lower NO production. There are many IGF-1 receptors in cardiac myocytes, vascular smooth muscle cells, and endothelial cells. IGF-1 diminished apoptosis in these cells, controlling their integrity (Sattler, 2013). These effects lead to a greater prevalence of developing cardiovascular diseases (Devin et al., 2008).

A GH deficiency in adults leads to changes in the lipid profile, including an increase in low-density lipoprotein (LDL) and triglycerides, and a decrease in high-density lipoprotein (HDL). Low levels of GH and IGF-1 have been associated in different cohorts with endothelial dysfunction, increase in carotid intima-media thickness (CIMT), impaired coronary blood flow, coronary artery disease, congestive heart failure, left ventricular hypertrophy, arterial hypertension, myocardial infarction, and ischemic stroke (Caicedo et al., 2018).

## 3 GH deficiency in adults

In adults, HGH deficiency is associated with changes in body composition, including reduced lean body tissue, skeletal and myocardial muscle, increased total and trunk fat mass (FM), osteopenia or osteoporosis, and dry, thin, wrinkled skin. HGH deficiency also disrupts metabolism, causing reductions in total protein synthesis, lipolysis, and fat oxidation; elevations in total and LDL cholesterol, fibrinogen, and PAI-1; and insulin resistance. Lastly, HGH deficiency has adverse implications in other functions such as cardiac stroke volume, oxygen-carrying capacity, skeletal muscle strength, aerobic exercise capacity, vitality, vigor, blood pressure, and quality of life in general (Sattler, 2013).

Numerous categories of adult patients are susceptible to HGH deficiency, encompassing individuals with a history of sellar mass lesions, pituitary surgery or radiotherapy, traumatic brain injury, subarachnoid hemorrhage, and childhood-onset HGH deficiency. Individuals in these categories typically face a risk of further deficiencies in pituitary hormones, except for certain patients with childhood-onset HGH deficiency, which may exist in isolation, either being idiopathic or of genetic origin. Conversely, isolated, idiopathic HGH deficiency that begins in adulthood is highly improbable (Tritos and Biller, 2021).

Correctly recognizing adult patients with GH deficiency is crucial, as they might gain from initiating HGH replacement therapy. The diagnosis of HGH deficiency relies on showing an absence of HGH release in response to various pharmacological agents that typically stimulate its secretion (Tritos and Biller, 2021). The last diagnostic criteria published in 2019 by the American Association of Clinical Endocrinologists (AACE) reported a cutoff point for GH after different stimuli tests including insulin with a cut-off of GH ≤ 5 mcg/L; glucagon with a cut-off of GH ≤ 1 or ≤3 mcg/L depending on their body mass index (BMI) ≤25 kg/m^2^ or >25 kg/m^2^, respectively; and macimorelin, with a cut off of GH ≤ 2.8 mcg/L (Yuen et al., 2019).

## 4 Evidence of growth hormone as a human anti-aging therapy

Aging, which exhibits a reduction in HGH levels and sensitivity of somatotrophs to the action of GHRH, leads to changes in body composition, muscle strength. Reducing muscle strength contributes to frailty and fracture risk in the older people (Taaffe et al., 1994). Repetitive administration of GHRH has been shown to restore the attenuated response in different clinical trials (Table 1).

**TABLE 1:** Clinical trials using GHRH.

Study	Patients	Regimen	Results
Güldner et al. (1997)	13 healthy adults (60–93 years)	4 × 50 µg GHRH iv vs. placebo	Reduction in nocturnal awakenings and increase in initial NREM sleep period
Khorram et al. (1997)	19 healthy adults (55–71 years)	10 μg/kg GHRH sc daily for 16 weeks vs. placebo for 4 weeks	Increase in ST in both genders and an increase in LBM, insulin sensitivity, general wellbeing, and libido in men
Khorram et al. (1997)	19 healthy adults (55–71 years)	10 μg/kg GHRH sc daily for 16 weeks vs. placebo for 4 weeks	Increase in lymphocytes expressing CD71, in monocytes, in B cells, in T cell receptor alpha/beta, and in T cell receptor gamma/delta
Vittone et al. (1997)	11 healthy men (64–76 years)	2 mg GHRH sc daily for 6 weeks	Two of six measures of muscle strength improved (upright row and shoulder press), and muscle endurance improved (abdominal crunch)
Perras et al. (1999)	23 healthy men (21–81 years)	300 µg of GHRH intranasally once vs. placebo	Increase of SWS and REM sleep
Antonijevic et al. (2000)	35 adults with depression (19–76 years) and matched controls	4 × 50 µg GHRH iv once vs. placebo	Enhance in non-REM sleep and the particular stage 2 sleep in men
Veldhuis et al. (2004)	22 healthy men (53–68 years)	1 mg vs. 4 mg of GHRH twice daily for 3 months	Both doses shorten the time required to walk 30 m and ascend 4 flights of stairs, and the higher dose increases TBW and FFM and reduces TAA
Vitiello et al. (2006)	89 healthy adults (60–85 years)	GHRH 1 mg/day for 6 months	Improvement in cognitive performance measured by WAIS-R performance IQ, WAIS-R picture arrangement, finding A’s, verbal sets, and single-dual task
Baker et al. (2012)	152 adults, 76 healthy vs. 61 with MCI (55–87 years)	Tesamorelin 1 mg/day sc for 20 weeks vs. placebo	Improvement in executive function, a reduction in percent body fat by 7.4%, and an increase fasting insulin levels
Friedman et al. (2013)	30 adults, 13 healthy vs. 17 with MCI (55–87 years)	Tesamorelin 1 mg/day sc for 20 weeks vs. placebo	Increase of GABA levels in all brain regions, increase of NAAG levels in the dorsolateral frontal cortex, and decrease in MI levels in the posterior cingulate

FFM, fat-free mass; GABA, γ-aminobutyric acid; iv, intravenously; MI, myo-inositol; NAAG, N-acetylaspartylglutamate; sc; subcutaneously; TAA, total abdominal adiposity; IQ, intelligence quotient; WAIS-R, wechsler adult intelligence scale; REM, rapid eye movement; ST, S; LBM, lean body mass; GHRH, growth-hormone releasing-hormone; SWS, slow wave sleep; TBW, total body water.

The administration of GHRH in healthy non-obese older men shows statistically significant elevated HGH levels and a dose-dependent increase of IGF-1 concentration, reversing their age-related declines (Corpas et al., 1992). GHRH treatment may increase muscle strength, altering the baseline relationship between muscle strength and muscle bioenergetics in a manner consistent with a reduced need for aerobic metabolism during exercise (Vittone et al., 1997). A nightly administration of GHRH analog in age-advanced men and women increased skin thickness (ST), lean body mass (LBM), IS, and general wellbeing (Khorram et al., 1997). In turn, it has been observed that administration of said analog (recombinant human GHRH-1,44-amide) in older men, shortened the time required to walk 30 m and ascend four flights of stairs. Also, with benefits in body composition in older men with a dose of 4 mg reaching an increase in the total body water (TBW) and fat-free mass (FFM), and a reduction in total abdominal adiposity (TAA) (Veldhuis et al., 2004).

In normal aging, a decline in sleep continuity, a decrease in slow wave sleep (SWS), an earlier nocturnal cortisol rise, and a blunted HGH secretion happen. The pulsatile administration of GHRH in healthy seniors significantly reduced nocturnal awakenings and increased the first non-rapid-eye-movement (NREM) sleep period (Güldner et al., 1997). Intranasal GHRH had shown a reduction in cortisol nadir concentrations at the beginning of sleep and a decrement in the sleep-induced elevation in HGH concentrations during early sleep. Moreover, results indicated that intranasal administration of GHRH increased rapid eye movement (REM) sleep and SWS, with this influence concentrating on the second half of sleep time. These effects may mimic the dual neuronal and endocrine function of hypothalamic GHRH activity (Perras et al., 1999). The pulsatile administration of GHRH promoted NREM sleep, stage 2 sleep, and sleep continuity, suggesting potential benefits for cognitive function, at least in men (Antonijevic et al., 2000).

With aging, there is a dynamic interplay between factors that lead to neurodegeneration and cognitive impairment and factors that lead to neuroplasticity and improved cognitive function (Murman, 2015). The treatment with GHRH compared to placebo in older adults showed an ameliorated cognitive decline independent of gender, estrogen status, and baseline cognitive capacity (Vitiello et al., 2006). In turn, it was seen that the administration of tesamorelin, a stabilized analog of GHRH, had favorable effects on the cognition of adults with mild cognitive impairment (MCI) and healthy older adults with a particular benefit on executive functions (Baker et al., 2012).

### 4.1 Administration of rHGH as treatment.

In addition to the effects seen with GHRH administration, beneficial effects have been seen with HGH administration in different clinical trials (Table 2). FFM and TBW increased with the administration of HGH in older adults (Veldhuis et al., 2004). Also, whole-body protein synthesis and breakdown rates are affected by the administration. In older men, an administration of HGH for 6 months increases LBM by an average of 4.3% and decreases FM by 13.1% (Papadakis et al., 1996). Also, it has been shown that HGH treatment for 3 months increases LBM, muscle mass, and thigh strength as evaluated by isokinetic dynamometry in healthy men over 60 (Welle et al., 1996; Harman and Blackman, 2004). An increase in strength was demonstrated in older adults (mean age 65.2 years, range 61–74 years) treated with HGH replacement therapy for 10 years at a mean initial dose of 0.72 mg/day, which was subsequently reduced to 0.37 mg/day. This resulted in a 108%–113% increase in knee flexor strength, a 95%–118% increase in knee extensor strength, and an 87%–93% increase in grip strength (Götherström et al., 2010).

**TABLE 2:** Clinical trials using rhGH.

Study	Patients	Doses	Results
Marcus et al. (1990)	18 healthy adults (67.7 ± 0.86 years)	0.03, 0.06, or 0.12 mg/kg rhGH daily for 7 days	Increase in IGF-1, PTH, and calcitriol levels, increase in urinary excretion of hydroxyproline and calcium, and decrease in urinary nitrogen and sodium excretion
Rudman et al. (1990)	45 healthy men (61–81 years)	0.03 mg/kg rhGH sc 3 times a week for 18 months vs. placebo	Increase in LBM, skin thickness, and muscle area, and decrease in adipose mass
Holloway et al. (1994)	27 healthy women (60–82 years)	0.043 mg/kg rhGH daily for 6 months vs. placebo	Decrease in fat mass by 11% and percent fat by 9%, and increase in LBM by 6.7%, and the markers of bone turnover
Yarasheski et al. (1995)	23 healthy and sedentary men (64–75 years)	12.5–24 μg/kg/day rhGH + resistance exercise for 16 weeks vs. placebo + resistance exercise	Increase in TBW, FFM, and whole-body protein synthesis, and decrease in fat mass
Papadakis et al. (1996)	52 healthy men (70–85 years)	0.03 mg/kg of rhGH 3 times per week for 6 months vs. placebo	Increase in LBM by 4.3%, decrease in fat mass by 13.1%, increase in Trails B score by 8.5 s
Welle et al., 1996	14 healthy adults (62–74 years)	0.03 mg/kg rhGH sc 3 times per week for 3 months	Increase in LBM, muscle mass, and thigh strength, and reduce whole body leucine oxidation by 36%
Butterfield et al. (1997)	14 healthy women (66–82 years)	0.025 mg/kg rhGH daily vs. 0.015, 0.03, or 0.06 mg/kg twice daily for 1 month	Increase in nitrogen balance, protein synthesis, and protein breakdown in both groups
Holloway et al. (1997)	84 osteopenic postmenopausal women (69.2 ± 6.5 years)	20 μg/kg rhGH daily for 7 days vs. placebo followed by 100 U/day salmon CT daily for 5 days vs. placebo followed by 44 days of supplemental calcium (56-day cycle) over 2 years	Increase in lumbar spine BMD, total hip BMD, and femoral trochanter BMD
Kozakowski et al. (1998)	18 healthy men (60.2 ± 2.4 years)	0.125 IU/kg/week rhGH daily sc for 12 months	Increase in lumbar spine BMD and femur neck BMD
Henrik et al. (2000)	16 healthy women (75 ± 1 year)	0.5 IU/m^2^ rhGH sc daily for the first week, 1 IU/m^2^ rhGH sc daily for the second week, and 1.5 IU/m^2^ rhGH sc daily until 12 weeks + endurance training program vs. placebo + endurance training program	Increase in muscle citrate synthase activity, muscle L-3-hydroxyacyl-CoA dehydrogenase activity, LBM, and decrease in fat mass
Taaffe et al. (2001)	27 obese postmenopausal women (59–79 years)	0.025 mg/kg rhGH, 0.015 mg/kg rhIGF-I, both, or placebo, daily for 12 weeks	Decrease in limb and trunk fat in all groups and a greater decrease in trunk fat compared to limb in the rhGH group
Münzer et al. (2001)	110 healthy adults (65–88 years)	20 μg/kg rhGH alone 3 times per week vs. HRT in women and T in men vs. both vs. placebo for 6 months	Increase in IGF-1 levels in the rhGH group, decrease total abdominal area in the rhGH + T group, decrease in subcutaneous fat in rhGH and rhGH + T groups
Harman and Blackman. (2004)	131 healthy adults (65–88 years)	rhGH vs. HRT in women and T in men vs. both vs. placebo for 26 weeks	Increase in IGF-1 levels in the rhGH and rhGH + HRT or T groups, increase in LBM in the rhGH and rhGH + HRT or T groups, decrease in fat mass in the rhGH and rhGH + HRT or T groups, increase in VO2 max by 8.5% in the rhGH + T group
Hameed et al. (2004)	19 healthy men (70–82 years)	0.5 IU/m^2^ rising to 1.5 IU/m^2^ rhGH or placebo with/without resistance training for 12 weeks	Increase of IGF-1Ea mRNA by 237% and MGF mRNA by 80% in the rhGH group, and increase of IGF-1Ea mRNA by 167% and MGF mRNA by 456% in the rhGH + resistance training group
Giannoulis et al. (2006)	80 healthy men (65–80 years)	rhGH (dose titrate to produce IGF-1 levels in the upper half of the age-specific reference range) vs. T vs. both vs. placebo	Increase in LBM in rhGH and rhGH + T groups, decrease in total body fat in rhGH + T group, increase in midthigh muscle and aerobic capacity in rhGH + T group, and increase in quality of life in rhGH and rhGH + T groups
Devin et al. (2008)	10 healthy adults (26–65 years)	0.03 mg/kg/week rhGH for 4 weeks, then 0.06 mg/kg/week for 4 more weeks	Increased IGF-1 levels and early-outgrowth EPCs
Giannoulis et al. (2008)	21 healthy men (65–75 years)	rhGH vs. T vs. both vs. placebo for 6 months	Increase in IGF-1 levels in rhGH and rhGH + T groups, increase in leucine rate of appearance and nonoxidative leucine disposal rate in rhGH and rhGH + T groups, increase in midthigh muscle mass and maximal oxygen capacity in rhGH + T group, and increase in androgen receptor expression in rhGH + T group
Münzer et al. (2009)	131 healthy older adults (65–88 years)	rhGH vs. HRT in women and T in men vs. both vs. placebo for 26 weeks	Increase in 120 min insulin and AUC insulin, and decrease of ISI and QUICKI in women of the rhGH group, increase in 120 min, AUC glucose, and AUC insulin in men of the rhGH and rhGH + T groups, decrease of ISI in men of the rhGH and rhGH + T groups, decrease of QUICKI in men of the rhGH group, decrease in LDL-c in rhGH groups, and increase in triglycerides in rhGH groups
Sattler et al. (2009)	122 healthy men (70.8 ± 4.2 years)	5 or 10 g/day T with 0, 3, or 5 μg/kg/day rhGH for 16 weeks	Increase in LBM, appendicular lean tissue, composite maximum voluntary strength of body muscles, and aerobic endurance, and decrease in total fat mass and trunk fat in all six groups
Doessing et al. (2010)	10 healthy sedentary men (30 ± 2 years)	33.3 μg/kg rhGH sc daily for 7 days, increased to 50 μg/kg/day on days 8–14	Increase IGF-1 serum levels and IGF-1 mRNA expression in tendon and muscle, and increase tendon collagen I mRNA expression by 3.9-fold, tendon collagen protein synthesis by 1.3-fold, muscle collagen I mRNA expression by 2.3-fold, and muscle collagen protein synthesis by 5.8-fold

AUC, area under the curve; BMD, bone mineral density; EPCs, endothelial progenitor cells; FFM, fat-free mass; HRT, hormone replace therapy; IGF-1, insulin-like growth factor 1; Im, intramuscular; ISI, insulin sensitivity index; LBM, lean body mass; MGF, mechano growth factor; mRNA, messenger ribonucleic acid; PTH, parathyroid hormone; QUICKI, quantitative insulin sensitivity check index; rhGH, recombinant human growth hormone; rhIGF-1, recombinant human insulin-like growth factor 1; T, testosterone; TBW, total body weight; Sc, subcutaneously.

The administration of HGH to healthy older adult men with IGF-I concentration less than 0.35 U/mL showed a mean plasma IGF-1 level rose into the youthful range of 0.5–1.5 U/mL and this effect was dose-dependent (Marcus et al., 1990; Rudman et al., 1990). With this range, there was a statistically significant increase in LBM, a decrease in adipose tissue, an increase in ST, and an increase in liver and spleen volumes (Rudman et al., 1990; Münzer et al., 2001). In the case of healthy older women, the administration of HGH resulted in a decrease in FM, an increase in markers of bone turnover (hydroxyproline, pyridinoline excretion, and osteocalcin), and a reduction of LDL-cholesterol (Holloway et al., 1997). The administration of HGH to older people significantly decreased urinary nitrogen and sodium excretions, while elevated serum estradiol and testosterone (Marcus et al., 1990). Also, LBM increased, and FM decreased statistically significantly with HGH treatment in older adults performing resistance training, compared to placebo (Taaffe et al., 1994). It has been observed that the administration of HGH caused an increase in the serum levels of HGH and IGF-I, as well as in the expression of IGF-I and collagen mRNAs in tendons and muscles. The collagen synthesis in the tendon and muscle suggests that HGH is more important in strengthening the matrix tissue than muscle cell hypertrophy in adult human musculotendinous tissue (Doessing et al., 2010). Another isolated effect observed with the administration of HGH in healthy adults is the augmentation of the early-outgrowth EPC population, having a role in maintaining vascular integrity (Devin et al., 2008).

The decline in HGH secretion and serum levels of IGF-1 during aging may be a causal factor in the development of osteopenia. The administration of HGH and IGF-I increased the whole body and muscle protein synthesis in older women (Butterfield et al., 1997). HGH administration increases osteocalcin concentration, besides bone mineral density (BMD) in the lumbar spine and femur neck (Lissett and Shalet, 2000). HGH replacement therapy in older men may be useful to protect against osteoporosis progression (Kozakowski et al., 1998). Clanget et al. (2001) observed an improvement in BMD in 12 patients with a mean age of 42.5 years (ranging from 24 to 61 years) following 6 years of HGH treatment with a mean daily dose of 2.4 IU. This increased BMD of 0.16 g/cm^2^ of the lumbar spine, represented a 15.9% improvement. Also, it was seen that HGH given cyclically achieved statistically significant increases in BMD of the lumbar spine and of some areas of the hip in postmenopausal women. Nevertheless, these gains are less marked than those achieved with estrogen or bisphosphonates, being associated with a relatively high incidence of adverse effects, including the development of peripheral edema and carpal tunnel syndrome (Holloway et al., 1997).

Furthermore, the administration of HGH has been observed to impact cognitive function positively. This phenomenon was investigated in a cohort of 34 patients, aged between 60 and 77 years, with a mean daily dose of 0.16 ± 0.06 mg for 12 months. The effects were evaluated at baseline and after weeks 24 and 52 using a computerized psychometric test package (Neurobehavioral Examination System-2) (Sathiavageeswaran et al., 2007). The findings indicated that the group treated with HGH exhibited statistically significant superior mean serial digit learning scores compared to the placebo group.

### 4.2 HGH combined therapies

There are combinations of therapies that have been tested together with the administration of HGH. One of them is its administration with endurance training in older women, which achieves a marked increase in muscle citrate synthase activity and an increase in muscle L-3-hydroxyacil-CoA dehydrogenase activity. Also, they showed a decrease in FM and an increase in LBM, translating both findings into effects on muscle oxidative enzymes and reduction in body fat (Henrik et al., 2000). Similar programs that combined the administration of HGH with exercise and/or moderate caloric restriction, had shown a decrease in central fat in older women and an increase in the proportion of type 3 fibers, which correlated with muscle strength in older subjects (Taaffe et al., 2001). Other effects seen in this combination are the increase of MGF mRNA and IGF-IE mRNA in older men, suggesting an enhancement of mechanical loading compared to resistance training alone (Hameed et al., 2004). Another combination that has been performed is the coadministration of HGH with testosterone which led to a greater increase in LBM, a decrease in total body fat, and an increase in midthigh muscle and aerobic capacity in healthy older men compared with HGH alone (Giannoulis et al., 2006). In another trial, the administration of HGH and testosterone enanthate accompanied by aerobic and anaerobic fitness had a statistically significant improvement in parameters such as FFM, total body fat, trunk fat, total LBM, appendicular lean tissue, total cholesterol, high-density lipoprotein cholesterol, low-density lipoprotein cholesterol, triglyceride, testosterone, IGF-1, HGH, leucine rate of appearance, nonoxidative leucine disposal rate, maximal oxygen uptake, anaerobic threshold, and maximal work rate (Giannoulis et al., 2008; Huang et al., 2005; Sattler et al., 2009).

### 4.3 Administration of other compounds with action on the HGH/IGF-1 axis

Other components can also simulate or alter the HGH/IGF-1 axis. Among them is growth hormone-releasing peptide 6 (GHRP-6), a synthetic compound that releases HGH. The combined administration of GHRH and GHRP-6 elicited a greater statistically significant HGH increase than GHRH alone or GHRP-6 alone. This indicates that the impaired HGH secretion in late adulthood is a functional and potentially reversible state (Micic et al., 1995).

Cholinergic enhancement by pyridostigmine (PD) has a stimulatory effect on HGH response to GHRH. PD probably reduces somatostatin release from the hypothalamus by increasing the central cholinergic tone. PD induces a significant increase in HGH secretion over the basal value and enhanced its response to GHRH. The effect of PD on HGH secretion suggests that cholinergic mechanisms may be involved in HGH control in normal aging (Giusti et al., 1991). The cholinergic stimulatory regulation of HGH release is reduced in older cycling women. Because acetylcholine inhibits hypothalamic somatostatin release, the reduced cholinergic tone in older subjects increases somatostatinergic tone. Normalization in older women of the reduced HGH response to GHRH, in combination with the administration of arginine (ARG) by inhibiting the somatostatinergic control, supports this hypothesis (Coiro et al., 2016). The responsiveness of somatotroph cells to GHRH is reduced in older humans but restored by ARG which most likely acts by inhibiting hypothalamic release of somatostatin. The combined administration of GHRH and ARG may be useful in restoring the impaired function of the HGH-IGF axis in aging (Ghigo et al., 1994; Iovino, et al., 1989). Donepezil, a cerebral selective cholinesterase inhibitor (ChEI) increased HGH and IGF-I levels in older males, demonstrating that age-related downregulation of this axis is reversed considerably (Obermayr et al., 2005).

Although both spontaneous and stimulated HGH secretion undergoes age-related decline, the secretory capacity of somatotroph cells is preserved in human aging. Hexarelin (HEX), a synthetic hexapeptide, has a strong and reproducible HGH-releasing activity after intravenous, subcutaneous, intranasal, or oral administration. It’s maximum effective dose releases more HGH than the maximal effective dose of GHRH in older subjects. The effect of HEX on HGH secretion is age-dependent, and the HGH response to the combined administration of HEX and GHRH is significantly higher in young subjects compared to older subjects. ARG does not potentiate the HGH response to HEX in young subjects, although it enhances significantly in older subjects (Arvat et al., 1994). Chronic but intermittent treatment with HEX, administered either by intranasal or oral route, does not desensitize the HGH response to the peptide. After HEX treatment a trend towards increase is shown for IGF-1 and insulin-like growth factor binding protein 3 (IGFBP-3) levels, strengthening the hypothesis that prolonged administration of HEX may restore the reduced HGH release in aging (Ghigo et al., 1996).

HGH induces lipolysis, increasing plasma FFA levels, and in turn, FFA levels can reduce HGH release. Old age is associated with a blunted HGH response to several stimuli, including GHRH and FFA. Acipimox, a nicotinic acid analog able to block lipolysis, enhances the HGH response to GHRH in older adults (Pontiroli et al., 1996). MK-677, a mimetic HGH-releasing peptide administered in older individuals enhanced pulsatile HGH release, increased serum HGH and IGF-I levels, and at a dose of 25 mg/day even restored serum IGF-I concentration (Chapman et al., 1996). In turn, aging in humans is accompanied by a progressive decline in the secretion of the adrenal androgens dehydroepiandrosterone (DHEA) and DHEA sulfate (DS), paralleling that of the HGH-IGF-I axis. Replacement of DHEA in adults and older adults induced an increase in the bioavailability of IGF-I, as well as improvements in physical and psychological wellbeing in both genders (Morales et al., 1994).

## 5 Adverse effects

Despite the use of HGH for indicated purposes, such as that of HGH deficiency, and that it was classified as a treatment with a satisfactory safety profile by the European Society of Pediatric Endocrinology, Growth Hormone Research Society, and Pediatric Endocrine Society, its administration is not advised for antiaging purposes in healthy older people (Grimberg and Allen, 2017). Nevertheless, the information for its adverse effects is limited. Among the information available on this subject are the adverse effects reported in the clinical trials used for this purpose, however, some of them failed to report this aspect in their results. Regarding the adverse effects seen in clinical trials using HGH, reports are consistent, with a wide variety of organs and systems affected, including gastrointestinal, hematological, hormonal, and musculoskeletal.

The most frequent adverse effects that have been reported are those involving the hormonal and musculoskeletal aspects. Among the most frequent hormonal symptoms, is fluid retention, which is usually resistant to diuretics and has been reported in a range of 11%–100% of patients in varying degrees (Marcus et al., 1990; Holloway et al., 1994; Taaffe et al., 1994; Papadakis et al., 1996; Bonello et al., 1996; Henrik et al., 2000; Taaffe et al., 2001; Münzer et al., 2001). Another adverse effect is mastalgia or even gynecomastia, reported in 7%–14% of patients (Rudman et al., 1990; Papadakis et al., 1996). Both fluid retention and breast symptoms tend to be more frequent in women, particularly in postmenopausal women (Taaffe et al., 2001). Although less frequently, an increase in blood pressure has been reported in 7% of patients, this is attributed to the salt and water retention and increases in plasma renin activity (Harman and Blackman, 2004). Finally, rhGH increases mean levels of serum glucose at fasting and 120-min time points on a standard glucose tolerance test and has even been attributed to the development of T2D especially in men in 3% of users (Bonello et al., 1996; Harman and Blackman, 2004). Now, concerning musculoskeletal adverse effects, the most frequent was the development of carpal tunnel syndrome, either unilateral or bilateral, in 7%–50% of patients and the presence of arthralgias in 14%–77% of the treated individuals (Amato et al., 2000; Bonello et al., 1996; Ghiron et al., 1995; Giannoulis et al., 2006; Harman and Blackman, 2004; Henrik et al., 2000; Holloway et al., 1994; Münzer et al., 2001; Papadakis et al., 1996; Rudman et al., 1990; Taaffe et al., 2001; Welle et al., 1996). Although less commonly reported, at least one clinical trial documented arthritis in 25% of women treated with HGH. Similarly, a single trial reported myalgias but did not specify their frequency (Henrik et al., 2000; Giannoulis et al., 2008).

Regarding gastrointestinal adverse effects, there have been reports of only bloating. This has been reported in up to 60% of patients who received the hormone. However, it has been reported that it is resolved 2 weeks after the end of treatment (Marcus et al., 1990; Ghiron et al., 1995). On the other hand, regarding hematological adverse effects, a significant reduction in hemoglobin and hematocrit was once again reported only in one of the clinical trials; however, the authors attributed this to blood loss during the protocol, secondary to multiple blood samples being taken for analysis, reaching 800 mL throughout the entire study (Marcus et al., 1990).

As for less reported adverse effects, there is a clinical trial in which facial flushing was reported in 12% of the subjects, numbness of the extremities in 57%, fatigue in 40%–71%, headache in 12%, and increased prostate-specific antigen in men; however, they were seen when administered in conjunction with testosterone at high doses (Ghiron et al., 1995; Henrik et al., 2000; Taaffe et al., 2001; Sattler et al., 2009).

In a long-term study (10 years follow-up), including 86 patients with adult-onset pituitary disease who received HGH replacement therapy, four patients died, one from renal carcinoma, one from omental cancer, one from pulmonary edema (probably secondary to an acute myocardial infarction), and a fourth from cerebral infarction. Moreover, two patients discontinued the study after 8 and 9.5 years, one due to a malignant bladder tumor combined with cerebral infarction and the other due to chronic lymphocytic leukemia. In addition, another four patients developed T2D between 1 and 6.5 years of treatment, and six additional patients developed hyperlipidemia between 5 and 8.5 years (Götherström et al., 2007). Although causal associations cannot be deduced from these findings, they raise possible associations to be addressed in subsequent trials with study designs guided by this objective.

It is worth mentioning that there have been reports of isolated cases in which bodybuilders developed long-term adverse effects, including squamous cell carcinoma in the lung and bilateral laryngoceles (Moor and Khan, 2005; Yen and Westover, 2022). While HGH itself does not necessarily initiate cancer, its role in accelerating tumor progression is well-documented, particularly in individuals with latent or predisposing conditions. This aligns with findings that recombinant HGH therapy increases the risk of secondary neoplasms, rather than *de novo* tumor formation (He et al., 2022). These observations underscore the potential oncogenic risks associated with exogenous HGH use, particularly in susceptible populations.

## 6 Conclusions

So far, the FDA has only approved the use of rhGH for adults with three indications: HGH deficiency due to pituitary tumors or their treatment, the muscle-wasting disease that comes with human immunodeficiency virus (HIV), and short bowel syndrome. Clinical trials using recombinant forms of somatotropic axis hormones as antiaging therapies, have been conducted during the last 40 years. Despite obtaining positive results mainly in body composition, carbohydrate, protein, and lipid metabolism, cognitive performance, and vascular endothelial repair, frequent short-term adverse effects have also been observed, such as fluid retention, gynecomastia, hyperglycemia, increased blood pressure, carpal tunnel syndrome, arthralgias, and myalgias. However, the most worrying are the long-term adverse effects that have been described, such as an increase in cardiovascular complications, the development of T2D, and different types of cancer, which is why its application in real life as an antiaging therapy continues to be controversial and requires further studies to define the stage in life in which it would be most useful, the optimal time of use, and ways of monitoring and preventing its adverse effects.
